# Electrical and Thermal and Self-Healing Properties of Graphene-Thermopolyurethane Flexible Conductive Films

**DOI:** 10.3390/nano10040753

**Published:** 2020-04-15

**Authors:** Ke Wang, Zhimin Zhou, Jiahao Zhang, Jinyuan Tang, Peiyu Wu, Yuehui Wang, Yuzhen Zhao, Yong Leng

**Affiliations:** 1 Zhongshan Institute, University of Electronic Science and Technology of China, Zhongshan 528402, Guangdong, China; wkzsedu@126.com (K.W.); zzmzsedu@126.com (Z.Z.); zjhzsedu@126.com (J.Z.); tjyzsedu@126.com (J.T.); wpyzsedu@126.com (P.W.); 2 School of Materials and Energy, University of Electronic Science and Technology of China, Chengdu 610054, China; 3 Department of Materials Science and Engineering, Tsinghua University, Beijing 100084, China; zhaoyz@mail.tsinghua.edu.cn; 4 Zhongshan Breathtex Speciality Material Co., Ltd., Zhongshan 528441, Guangdong, China; lybreathtex@126.com

**Keywords:** graphene, thermopolyurethane, flexible conductive film, self-healing, electro-thermal response, infrared light thermal response

## Abstract

We fabricated graphene-thermopolyurethane (G-TPU) flexible conductive film by a blending method and systematically investigated the electrical, thermal and self-healing properties of the G-TPU flexible conductive film by infrared light and electricity. The experimental results demonstrate that the G-TPU composite films have good conductivity and thermal conductivity in the appropriate mass content of graphene in the composite film. The composite films have the good electro-thermal and infrared light thermal response performances and electro-thermal response performance is closely related to the mass content of graphene in the composite film, but the infrared light thermal response performance is not. The scratch on the composite film can be completely healed, using electricity or infrared light. The healing efficiency of the composite film healed using infrared light is higher than that of using the electricity, while the healing time of the composite film is shorter. Regardless of the self-healing method, the temperature of the self-healing is a very important factor. The self-healing conductive composite film still exhibits a good conductivity.

## 1. Introduction 

Thermoplastic polyurethane (TPU) films have been used widely in many fields, such as electronics, medical treatment, food and other industries. This is largely due to their excellent comprehensive properties, such as high strength, high toughness, abrasion resistance and oil resistance, and good processing performance [1,2,3,4]. However, they are susceptible to failure arising from micro-cracks generated on the surface and inside inner layer during processing and utilization, which lead to a sharp decrease in their sustainability, safety, and lifetime [5,6,7,8,9]. To solve this problem, self-healing polymers and composites that can heal themselves spontaneously and automatically in response to damage and regenerate its original structure and function, have gradually become a research hotspot [8,9,10,11,12,13,14,15]. Self-healing materials are new smart materials that can imitate the self-healing of organism after damage. That is, through the material or energy supply mechanism, the damaged part of the material can be automatically healed to improve the safety, lifetime, and environmental impact of man-made materials [16,17,18,19,20,21]. To obtain self-healing materials, many strategies have been explored, such as micro-containers containing healing agent and microvascular networks in the matrix, dynamic chemical bonds or weak interaction [22,23,24,25,26,27]. These self-healing materials can realize self-healing by using a large number of healing agents [28,29]. In theory, they can realize multiple self-healing of the damaged materials with the assistance of external light, heat, and electric field, et al. Although, great progress has been made in the research of the self-healing materials and techniques, there are still some critical problems that restrict their applications, such as low mechanical properties after healing, high cost, time-consuming, and low efficiency. Therefore, it is significant to fabricate a self-healing polymer and composites based on new materials, and then explore their potential applications, as well as new healing method. 

Graphene (G), a novel two-dimensional material, possesses large specific surface area, superior mechanical property, as well as being an excellent conductor of both heat and electricity, and having a good microwave and infrared absorbing capacity, so they have strong response to electricity and electromagnetic waves and infrared radiation (IR) [30,31,32,33,34]. Graphene is often used as fillers in preparing functional composites. Based on these properties, researchers fabricated graphene-based self-healing polymer composites by introducing graphene into the polymer [34,35]. Kim et al. reported, for the first time, self-healable PU nanocomposites, containing modified graphene by the near IR absorption of graphene [36]. Huang et al. prepared TPU containing few layers of graphene, and their healing performance by microwave radiation [5]. Luan et al. reported on the healing performance of TPU composites, filled with graphene-CNTs, under microwave radiation [6]. Oh et al. synthesized thermally self-healable graphene nanoplate-PU composites using in-situ Diels-Alder (DA) reaction of graphene nanoplate and furfuryl derivative as a chain extender of PU prepolymers [7]. Previous studies demonstrated that appropriate polymers, containing graphene, might develop some self-healable materials [30,31,32,33,34,35,36]. 

Most of the previous research work, relating to the polymer composites containing graphene, mainly focus on improving the mechanical property and self-healed performance by near IR light [5,6,30,31,32,33,34,35]. To the best of our knowledge, there are few reports that systemically investigate graphene-polymer composites as self-healing flexible conductive film. In this paper, we fabricated graphene-thermopolyurethane (G-TPU) flexible conductive film and systematically investigated the electrical and thermal and self-healing properties of G-TPU flexible conductive film. 

## 2. Experimental Approach 

### 2.1. Materials 

Graphene (TNERGO-10) with 1–2 layers and an average particle size of 8–15 μm and a purity of >98 wt% was purchased from Chengdu Institute of Organic Chemistry of the Chinese Academy of Sciences. *N*,*N*-Dimethylformamide (DMF) was purchased from Guangzhou Chemical Reagent Co., Ltd., Guangzhou, Guangdong, China; thermopolyurethane masterbatch (TPU) was purchased from Shenzhen Hongxin Plastic Material Technology Co., Ltd., Shenzhen, Guangdong, China. All the chemicals were used as received. 

### 2.2. Preparation of G-TPU Composite Film

According to the experimental formula, a certain amount TPU masterbatch was dissolved in 500 mL DMF. A formulated amount of graphene was added into a flask containing DMF solution under vigorous stirring and ultrasound for 30 min. Then, the formulated TPU solution was added into the above graphene DMF under vigorous agitation. The mixed solution was dispersed by a high-speed shear disperser for 60 min. Finally, the mixed solution was poured into the Teflon mould and dried at 70 °C until the weight did not change any more, then graphene- TPU (G-TPU) composite film were obtained. G-TPU composite film was peeled off for further testing.

### 2.3. Characterization

Differential scanning calorimetry (DSC) and thermogravimetric (TG) analysis were conducted via simultaneous differential thermal analysis (STA449F5, NETZSCH-Gertebau GmbH, Selb, Germany). Scanning electron microscope (SEM, Zeiss sigma 500, Carl Zeiss, Germany), atomic force microscopy (Dimension Edge, Bruker, America), and optical microscope (Nikon LV100, Nikon Co., Ltd., Japan) with a digital camera were used to investigate the microstructure of G-TPU composite film. Six different areas of the surface of sample were selected to obtain root mean square roughness (RMS) value and calculated as average value. The resistance was measured by a four-point probe system (ST2253, Suzhou Jingge Electronics Co., Ltd. Suzhou, Zhejiang, China). The resistance of each sample were each measured at twenty different sites and calculated from the average value of those measurements. An infrared thermal imager (UTI160G, range: −20–350 °C, accuracy: ±2 °C, UNI-T China Co., Ltd., Shenzhen, Guangdong, China). The thermal conductivity of sample was measured by a DRL-III heat flow meter instrument (Xiangtan Xiangyi Instrument Co. Ltd., Xiangtan, Hunan, China) according to the standard ASTM D5470. 

Mechanical measurement: tensile strengths of the sample before and after healed were measured by a universal tensile testing machine (UTM500, Shenzhen Sansi Zongheng Technology Co. LTD, Shenzhen, Guangdong, China). The extension rate was 50 mm/min. and the samples were cut into strips of 45 mm × 10 mm × 0.16 mm using a scalpel and then tested. 

Healing measurement: for the IR light healing, infrared lamp (PHILIP PAR38E, 250W, 0.76–5 μm, Royal Philips Electronics Co., Ltd, Suzhou, Jiangsu, China) was used as the light source; for the electrical healing, composite film was made by attaching the two ends of the film to two electrodes (clips coated with copper foil). A direct current voltage was supplied by a power supply (GPD-3303s, Suzhou Guwei electronics Co., Ltd., Suzhou, China) to the film through those two clips coated with copper foil that contacted the film edges. Before healing, a 5 mm scratch on the surface of composite film was cut by a razor blade, and then exposed in the environment with IR light and electricity. After that, the tensile strengths of the healed samples were tested again. The healing efficiency was calculated as the ratio of tensile strength of the healed sample and the virgin specimen. Eight samples were tested for each type in the experiment.

## 3. Results and Discussion

### 3.1. Electrical Property of G-TPU Film

The previous research work, relating to the PU composites filled with graphene, mainly focus on the effect of morphology (number of layers, size) and mass content of graphene, preparation method of graphene, and PU composites on polyurethane properties [5,6,30,31,32,33,34,35]. Few studies in the literature have reported on the effect of the initial concentration of TPU solution on the properties of the G-TPU film. However, the initial concentration of the TPU solution affects the viscosity of the solution, which affect the dispersion of graphene powders in the TPU solution, thereby, affecting the performance of the G-TPU composite film. Here, we prepared TPU solutions with the initial concentrations of 10 wt%, 20 wt%, and 30 wt%, respectively, and added graphene into the TPU solution according to the mass content of graphene in the G-TPU composite film of 0.5 wt%, 1.0 wt%, 1.5 wt%, 2.0 wt%, 3.0 wt%, 4.0 wt%, 5 wt%, and 7 wt%, respectively. 

**Figure 1:**
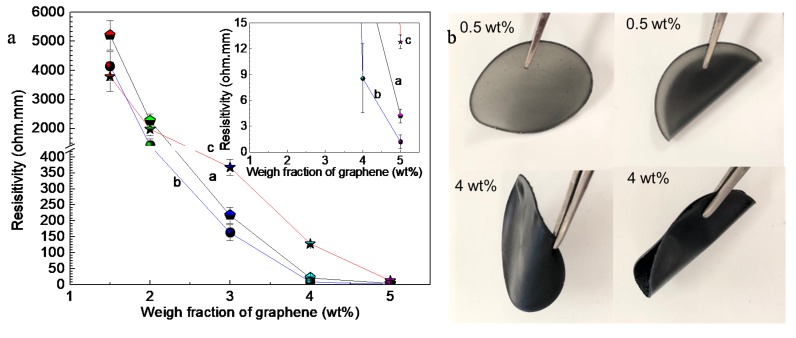
(**a**) Resistivity of G-TPU composite films with different mass contents of graphene and TPU initial concentrations of 10 wt% (curve a), 20 wt% (curve b), and 30 wt% (curve c), respectively, and (**b**) photos of samples with the mass content of graphene of 0.5 wt%, and 4.0 wt%, respectively, and initial TPU concentration of 20%.

Figure 1a shows the resistivity of G-TPU composite films with different mass contents of graphene and TPU solutions with initial concentrations of 10 wt% (curve a), 20 wt% (curve b) and 30 wt% (curve c), respectively, and Figure 1b shows photos of samples, prepared with 20 wt% PU and the mass content of graphene of 0.5 wt%, and 4.0 wt%, respectively. The inset in Figure 1a is local magnification. In our experiment, we found that when the mass content of graphene in the G-TPU composite film was lower than 1.5 wt%, none of the three composite films (three kinds of the initial concentrations of TPU) conducted electricity. In addition, when the mass content of graphene reached to 7.0 wt%, the mixed slurry was hard to stir by a high-speed shear disperser and to form film in the Teflon mould, so there are no data shown in Figure 1. When the graphene mass content is 1.5 wt%, the three composite films are all electrically conductive. The resistivity of G-TPU composite films fabricated with the initial TPU concentrations of 10 wt%, 20 wt%, and 30 wt%, respectively, are 5210.3 Ω∙mm, 4145.6 Ω∙mm, and 3790.1 Ω∙mm, respectively. As the graphene mass content increases, the conductivity gradually increases, and when the graphene content is 5 wt%, the resistivity of G-TPU composite films, fabricated with the initial TPU concentrations of 10 wt%, 20 wt%, and 30 wt% respectively, are 4.2 Ω∙mm, 1.19 Ω∙mm, and 12.8 Ω∙mm, respectively. During heat treatment of the sample, as the solvent evaporated, the crosslinking reaction is carried out in the composite film, and the resin matrix continued to shrink, making the graphene sheets more tightly overlap and stacked. The higher the graphene mass content, the better the conductive network. As shown in Figure 1a, when the TPU initial concentration is 20 wt%, the conductivity of the composite film is the best. The lower the initial concentration of TPU solution, the lower the viscosity of the TPU solution, which is not conducive to the formation of a compact stacking and overlapping structure of graphene sheets in the composite film. However, the higher the initial concentration of the TPU solution, the greater the viscosity of the TPU solution, resulting in the difficulty of dispersion of graphene in the TPU solution and the formation of aggregates of graphene. Additionally, this is related to the large specific surface area of graphene. As can be seen from Figure 1b, the composite films are flexible and the composite film with 0.5 wt% graphene still has a certain transparency, while the composite film with 4 wt% graphene is black and opaque. Based on the experimental results on the conductivity of the composite film, samples with the initial TPU concentration of 20% were selected for further experiments.

**Figure 2:**
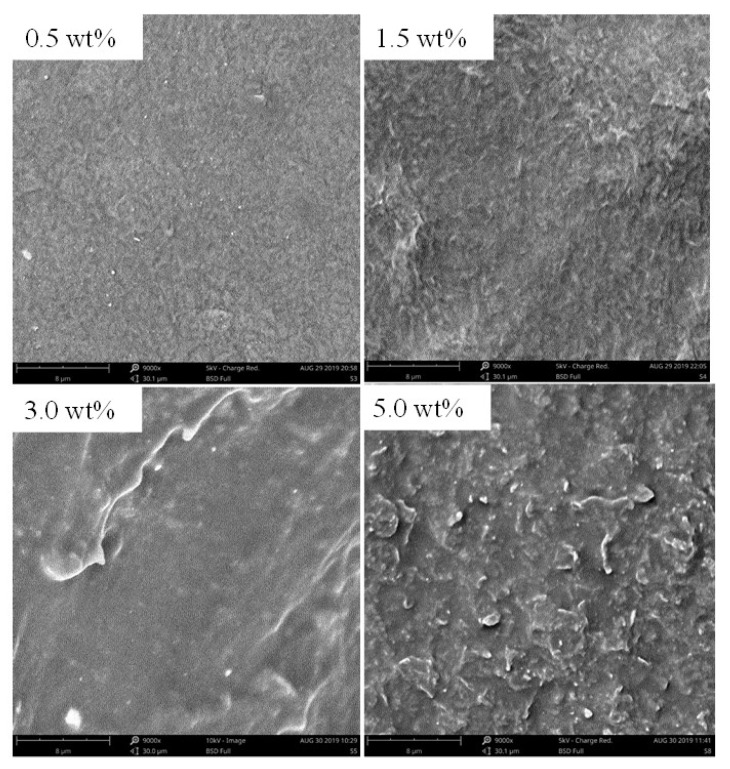
SEM images of surface morphology of the composite films with different mass contents of graphene.

Figure 2 shows SEM images of surface morphology of the composite films with the mass content of graphene of 0.5 wt%, 1.5 wt%, 3.0 wt%, and 5.0 wt%, respectively. The SEM image and Raman spectrum of graphene, in Figure S1, show supporting Information In the low mass content of graphene (Figure 2, 0.5 wt%, 1.5 wt%), the composite films displayed the TPU support strips. The composite films reveal rough surface characteristics and the surface roughness increases with the increase of graphene mass content. It is clearly that the graphene sheets are buried in the TPU matrix and exhibit stacked together, which is advantageous to the formation of electrically conductive pathways at proper concentrations (1.5 wt%–4.0 wt%). When the mass content of graphene in the composite film is 5.0 wt%, the obvious particles-like agglomerates on the surface of the composite film are observed, which may be formed by some of graphene agglomerates. 

**Figure 3:**
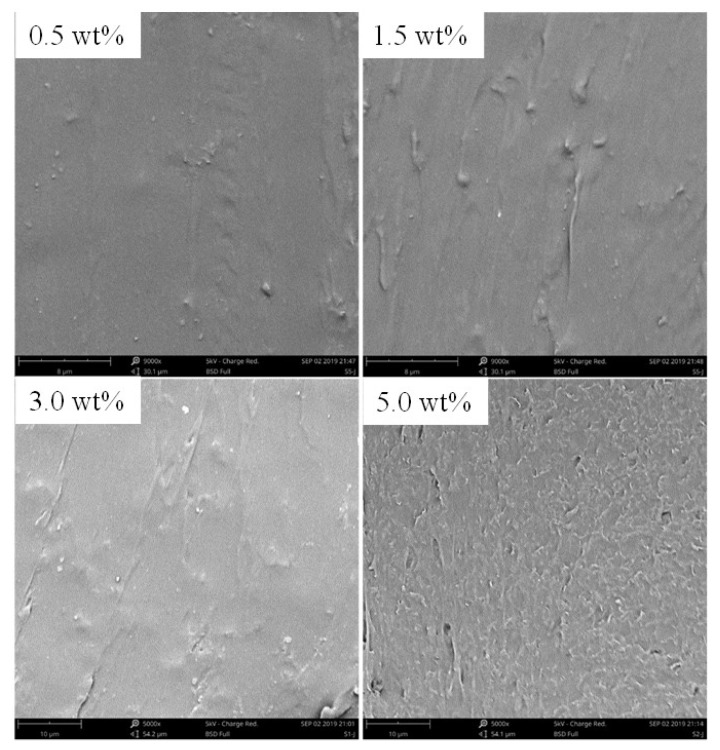
SEM images of the cross-section morphology of the composite films with different mass contents of graphene.

Figure 3 shows SEM images of the cross-section morphology of the composite films with the mass content of graphene of 0.5 wt%, 1.5 wt%, 3.0 wt%, and 5.0 wt%, respectively. In the cross-section view surface of composite films, it clearly shows a smooth surface, coated with TPU in the low mass content of graphene. The graphene dispersed and overlapped in the TPU matrix can been observed in the high mass content of graphene. It indicates a good compatibility between the fillers and matrix, which made closer contacts between them and promoted to form some conductive pathways and build conductive networks. However, when the mass content of graphene increases to 5.0 wt%, graphene tends to agglomerate in the polymer samples.

**Figure 4:**
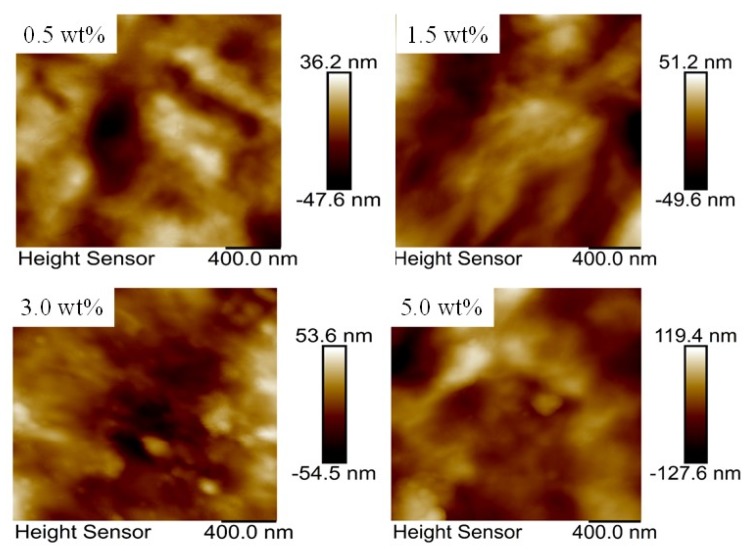
AFM images of the composite films with the mass content of graphene of 0.5 wt%, 1.5 wt%, 3.0 wt%, and 5.0 wt%, respectively.

Figure 4 shows AFM images of the composite films with the mass content of graphene of 0.5 wt%, 1.5 wt%, 3.0 wt%, and 5.0 wt%, respectively. The root mean square roughness (RMS) values of the composite films are 12.5, 23.5, 37.7, and 56.9 nm, respectively, showing that the surface roughness of the composite films increases as the increase of the mass content of graphene. 

### 3.2. Thermal Properties of G-TPU Films

**Figure 5:**
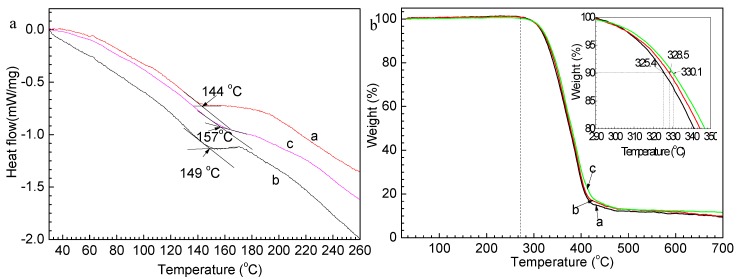
DSC (**a**) and TG (**b**) measurements of pure TPU (curve a), composites films containing graphene of 0.5 wt% (curve b) and 1.5 wt% (curve c).

In order to discuss the effect of graphene on the thermal property of G-TPU, the thermal profiles of the pure TPU matrix and the G-TPU were analyzed by DSC and TG. Figure 5 shows DSC (a) and TG (b) measurements of pure TPU (curve a in Figure 5), composites films containing the graphene of 0.5 wt% (curve b in Figure 5) and 1.5 wt% (curve c in Figure 5). As demonstrated in Figure 5a, the crystalline melting of TPU, which is from hard segment domains of the pure TPU, is at around 144 °C. After added graphene of 0.5 wt%, and 1.5 wt%, respectively, the crystalline melting of TPU moved to 149 °C, and 157 °C, respectively, indicating that graphene improves the thermal stability of TPU. The temperatures of weight loss of 10% of the TPU, composites films containing 0.5 wt% and 1.5 wt% graphene are 325.4 °C, 328.5 °C, and 330.1 °C, respectively, showing that composite films have good resistance to heat. This feature of thermal stability is consistent with that of DSC.

**Figure 6:**
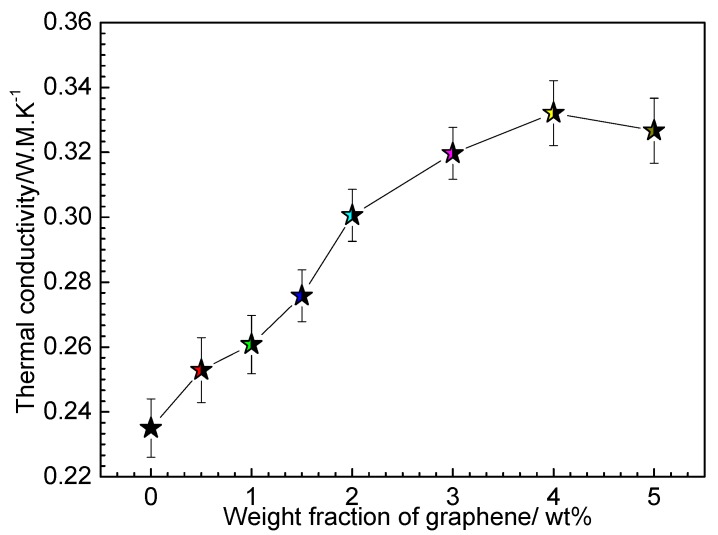
Thermal conductivity of G-TPU composite films with different mass contents of graphene.

Thermal conductivity of the composite film is another important property, so we measured the thermal conductivity of the G-TPU composite films with different mass content of gaphene and the initial TPU concentrations of 20 wt%, as shown in Figure 6. The thermal conductivity of the pure TPU is about 0.235 W·m^−1^·K^−1^ and the thermal conductivity of the composite film first increases and then decreases with the increase in mass content of graphene in composite film, as expected. When the graphene mass content reaches 4 wt%, the thermal conductivity of composite film reaches a maximum of 0.332 W·m^−1^·K^−1^, increasing by a factor of 1.4.

### 3.3. Electro-Thermal Response and IR Thermal Response Performances of G-TPU Films

**Figure 7:**
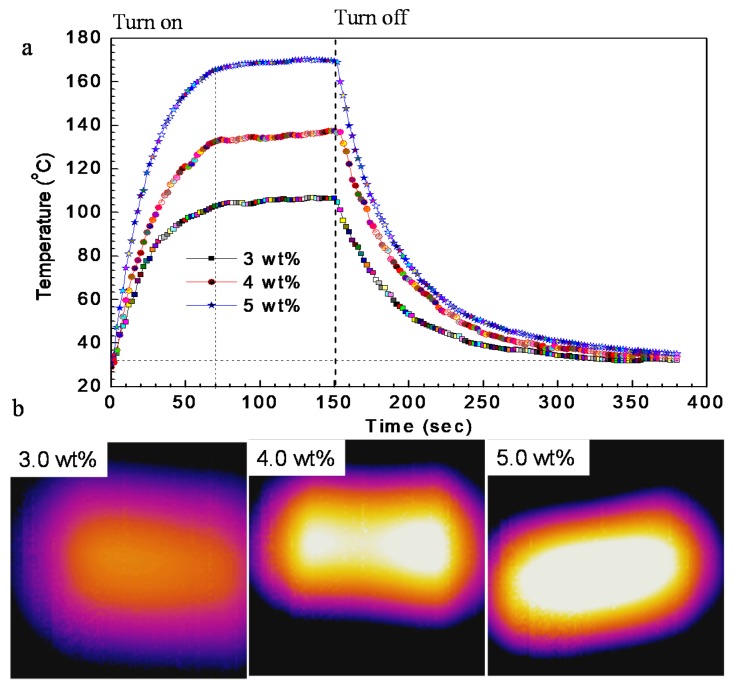
(**a**) Time-dependent temperature of the composite films with different mass contents of graphene under the operation for input voltage 15 V, (**b**) IR images of the composite films at steady state temperature.

Before studying the self-healing of the G-TPU composite films via electricity and IR light, we studied the electro-thermal response and IR thermal response performances of the G-TPU composite films [5,6,7,31,32,33,34,35]. The electro-thermal response performance of the composite films were studied by applying direct current to the composite films in a laboratory environment. The composite film was made using two clips coated with copper foil that contacted the film edges as electrodes. Figure 7a shows the time-dependent temperature of the G-TPU composite films, with different mass contents of graphene, under the operation for input voltage 15 V. The input voltage was turned on for 150 s and then turned off. The temperature response of the composite film was measured by an infrared camera and recorded every two seconds. The experimental results show that the composite films reached a steady state temperature from room temperature in 70 s, demonstrating fast electro-thermal response of the G-TPU composite film. The maximum steady state temperature of the G-TPU composite films with the mass content of graphene of 3.0 wt%, 4.0 wt%, and 5.0 wt%, respectively, was 106.4 °C, 137.2 °C, and 169.3 °C, respectively, showing that the efficient transduction of electrical energy into Joule heating is caused by the good conductivity of the composite film. It is worth pointing out that the good electro-thermal response performance of the composite film provides a possibility for self-healing of composite film via electricity. Additionally, the temperature distribution was measured by infrared images, as shown in Figure 7b. The infrared images clearly exhibit uniform color distribution over the composite film, indicating the uniform temperature distribution across the composite film. 

**Figure 8:**
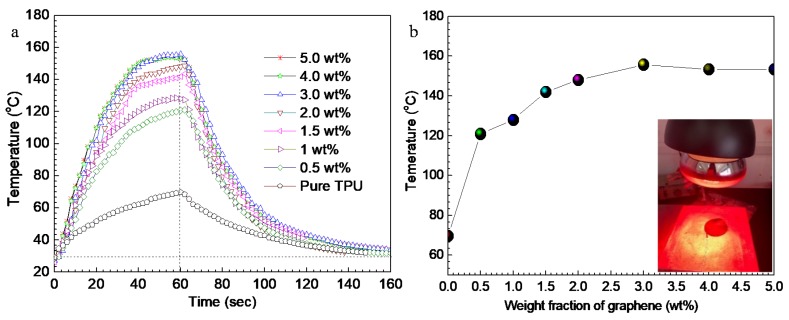
(**a**) Time-dependent temperature of the composite films with different mass contents of graphene via IR light, and (**b**) maximum temperature of the composite film heated for 60 s via IR light- dependent the mass content of graphene. The inset in (a) shows photo of the tested sample.

The IR thermal response performance of the G-TPU composite film was studied by applying IR light to the composite film. The G-TPU composite film was positioned under an IR lamp. Figure 8 shows the time-dependent temperature of the G-TPU composite films with different mass contents of graphene under the operation of IR lamp (Figure 8a) and maximum temperature - dependent the mass content of graphene (Figure 8b). The inset in (Figure 8a) shows photo of the tested sample. The IR light was turned on for 60 s and turned off. Comparison with Figure 7a, the time-dependent temperature curve of the IR thermal response performance in Figure 8a is a little bit different. The temperature of the G-TPU composite film first increases sharply and then slowly increases after 40 s as the IR light illumination time increases. With the increase of IR light illumination time. The temperature of the pure TPU film almost increases linearly and reaches to 69.7 °C after heated for 60 s. While, the temperature of the composite film with the mass content of graphene of 0.5 wt% reach to 120.9 °C after heated for 60 s, increased by 1.7 times. Meanwhile, we can see that maximum temperature of the G-TPU composite film heated for 60 s via IR light gradually increases as the mass content of graphene increases until the mass content of graphene reaches 3 wt%. We can see from the above experimental results that the graphene remarkably improved the IR response performance of TPU film. Due to its unique two-dimensional conjugated structure, graphene has a strong ability to absorb (near) IR light. As irradiated by IR light, graphene absorbs IR light energy and converts into heat and further transfer to the TPU matrix due to its good thermal conductivity, which increases the composite film temperature. However, it can be seen from the experimental results that the temperature of the composite film did not increase with the increase of the mass content of graphene, indicating that the absorption and conversion of graphene to infrared light reached saturation. We also measured the G-TPU composite films with the mass content of graphene of 0.5 wt% and 4.0 wt% and the initial TPU concentration of 30 wt% (Supporting Information, Figure S2). The time-dependent temperature of the G-TPU composite film was also independent of the initial concentration of TPU. The possible reason for this phenomenon is related to the distribution of graphene. 

**Figure 9:**
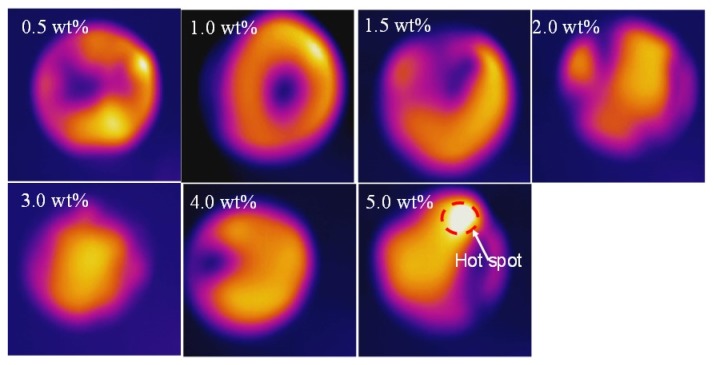
Infrared images of the composite films with different mass contents of graphene via IR light.

Figure 9 shows the IR images of the corresponding samples in Figure 7. The IR images show non-uniform color distribution, especially in the low mass content of graphene, indicating that non-uniform graphene distribution over the composite film. Aggregates of garaphene often causes “hot-spots”, as can be seen in Figure 9 (5.0 wt% of graphene). 

### 3.4. Self-Healing of G-TPU Films 

**Figure 10:**
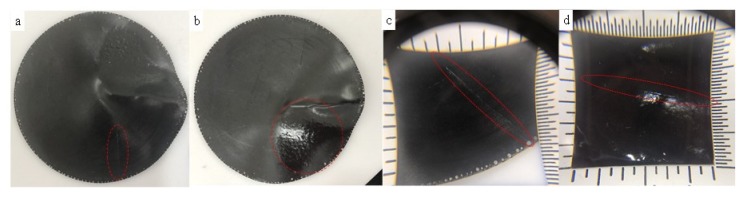
Photos (**a**) and (**b**) and photos in magnifying glass (**c**) and (**d**) of the composite film before (a) and (c) and after (b) and (d) healed using IR light.

The self-healing performances of the G-TPU composite film were studied by using electricity and IR light, respectively. Figure 10 displays photos (Figure 10a,b) and photos in magnifying glass (Figure 10c,d) of the G-TPU composite film before (Figure 10a,c) and after (Figure 10b,d) healed using IR light. Before healed, there is a scratch on the surface of the sample, but after healed, the scratch disappeared, indicating that self-healing behavior occurred. Additionally, the zone around the scratch of the self-healing sample appears brighter, which is obviously caused by hot - melting and solidification.

**Figure 11:**
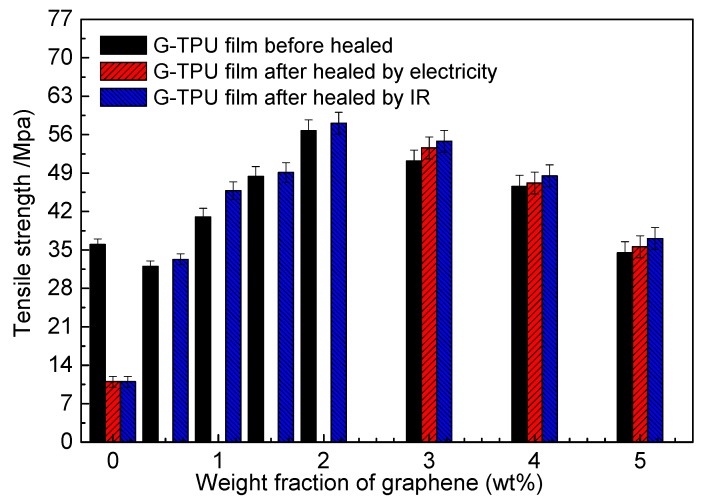
Tensile strength of G-TPU composite films before and after healed by electricity and IR light, respectively.

Graphene is often regarded as an excellent reinforcing filler for the composites due to its outstanding mechanical performance [8,9,10,11,12]. Researchers applied the ratio of tensile strength of the sample after and before healed to characterize the self-healing efficiency [8,9,10,11,12,13,14,15]. We measured the tensile strength of samples, including G-TPU composite films before, and after, healing by electricity, and IR light, respectively, as shown in Figure 11. For the IR light healing, all of scratch samples were positioned under an IR lamp for 5 min and kept the temperature of the composite film constant at 130 °C; For the electrical healing, all of scratch samples was treated at 20 V for 15 min. It should be noted that the composite film with the mass content of graphene less than 3% did not be used to heal by electricity because the sample is nonconductive or the resistance is too high, there is almost no any change in temperature. 

It is found that the mechanical property of composition film deteriorated as the mass content of graphene of 0.5 wt%, which was caused by non-uniform distribution of graphene in TPU (shown in Figure 1b), leading to local stress concentration. As the mass content of graphene in composite film increase, the mechanical property of film increase, and achieves to the highest when the mass content of graphene of 2.0 wt%. In the aspect of self-healing of the composite film, seen from Figure 10, the tensile strength of the self-healing composite films exceeds the value of the composite films, indicating full self-healing performance. The healing efficiency using IR light is higher than that of the electricity and the healing time is shorter than that of electricity. The experimental results demonstrate that self-healing by IR light is a better method than electricity as it works well in any samples with graphene, and the healing efficiency is only related to IR light power density delivered to the sample, the self-healing time, and the mass content of graphene. However, the healing efficiency is suitable for the self – healing of micro - crack in the conductive film.

**Figure 12:**
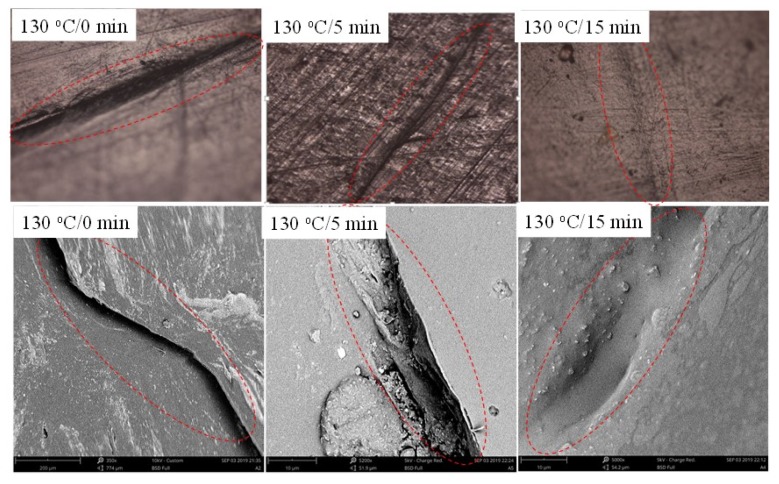
Optical and SEM images of scratch sample healed at 130 °C for different time using electricity.

Figure 12 displays the optical and SEM images of scratch sample healed at 130 °C for different time using electricity. Seen from Figure 11, the crack in composite film gradually decreases with the increase of the healing time and faint traces can be seen under the optical microscope and SEM after healed 15 min.

**Figure 13:**
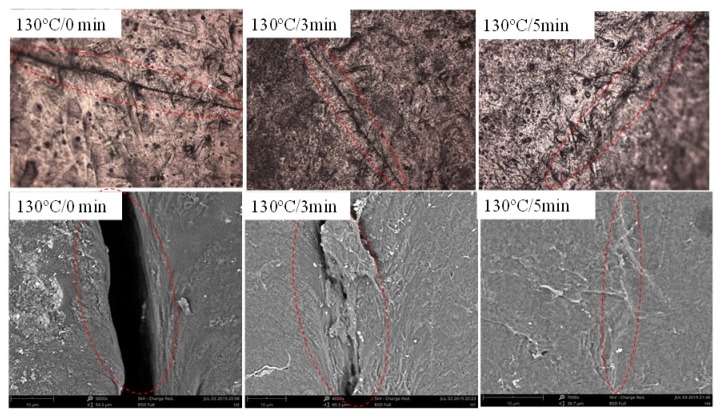
Optical and SEM images of scratch sample healed at 130 °C for different time using IR light.

Figure 13 displays the optical and SEM images of scratch sample healed at 130 °C for different time using IR light. Seen from Figure 12, the crack in composite film gradually decreases with the increase of the healing time and disappears after healing 5 min. Compared with Figure 12, the trace of the healed crack almost disappears, indicating that the self-healing performance is better. 

**Figure 14:**
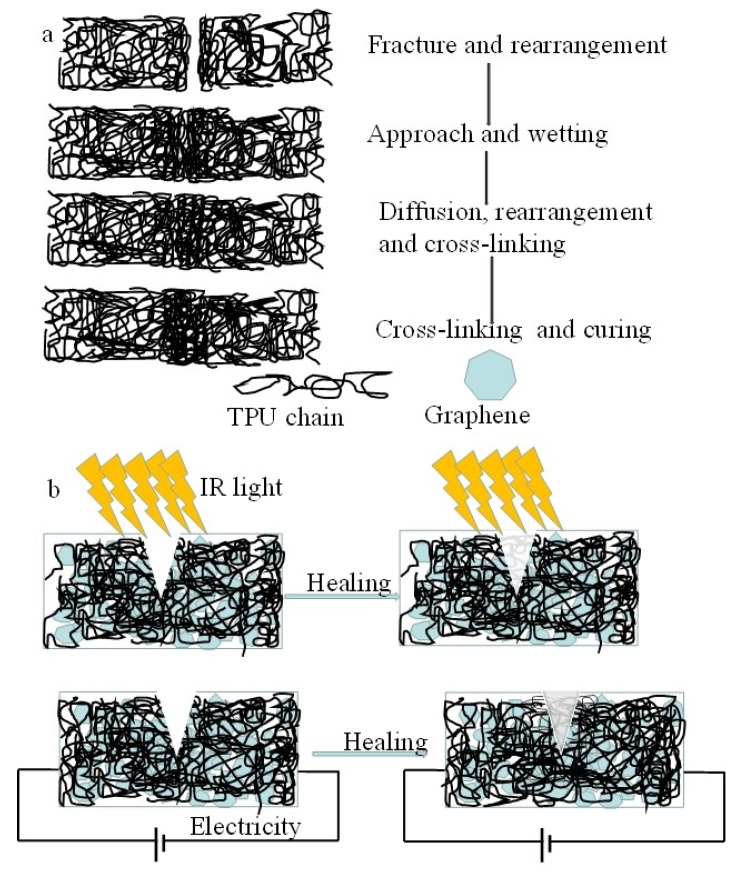
(**a**) crack healing theory and (**b**) schematic diagram of self-healing of the composite film by IR light and electricity, respectively.

According to the crack healing theory, the crack healing process of thermoplastic polymer mainly includes a rearrangement of a surface, the approach of a surface, wetting of a surface, low level diffusion between surfaces, diffusion and equilibration and randomization [5,32,33,34,35,36], as shown in Figure 14a. When the temperature of thermoplastic polymer is higher than glass transition temperature when heated, the self-healing process is accomplished by the wetting, diffusion, rearrangement of the thermoplastic polymer chains and the formation of a semi-interpenetrating network structure through the interpenetrating bridge at the crack section [5,32]. In our experiments, due to the excellent IR absorption and energy conversion and transfer of graphene, the temperature of G-TPU composite film rose to nearly the softening point in a short time, so the self-healing of the composite film can be obtained by the wetting, diffusion, rearrangement and cross-linking of TPU chains, and curing processes (Figure 14a). In the process of the self-healing composite film by electricity, due to the electrical energy transfer into Joule heating, the temperature of the composite film also increases, which promote the self-healing behavior of the composite film (Figure 14b). However, in the process of the self-healing composite film by IR irradiation, due to uniform and rapid distribution of heat and direct irradiation to the crack, iIt is conducive to the self-healing behavior of the composite film; on the other hand, the heat distribution of electricity process is dispersed and the heat starts from the bottom of the crack, so it takes long to achieve complete self-healing of the scratch. The closer the temperature of the thermoplastic polymer is to the softening point temperature, the easier it is for the above process to occur in a certain period of time, in order to obtain self-healing of the thermoplastic polymer in the short time, the composite film was not healed by electricity when the healing temperature at 110 °C and 120 °C for 15 min (Supporting Information Figure S3). We believe that regardless of the self-healing method, the temperature of the self-healing is a very important factor.

**Figure 15:**
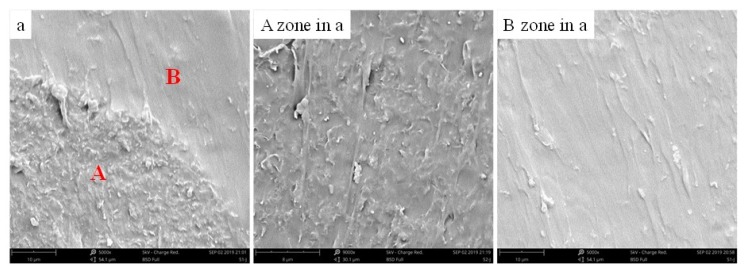
Cross-sectional SEM images of the self-healing composite film, A zone is the zone without crack, and B zone is the zone of self-healing of scratch.

To further demonstrate our discussion, we provided the cross-section SEM images of the scratch composite film, as shown in Figure 15. Part A is the zone without crack, and part B is the zone of self-healing of scratch. It is clearly that A zone where graphene is coated with TPU, showing a rough microstructure, however, more dense TPU and the smooth microstructure can be seen in B zone, indicating diffusion and rearrangement of the TPU chains in B.

**Figure 16:**
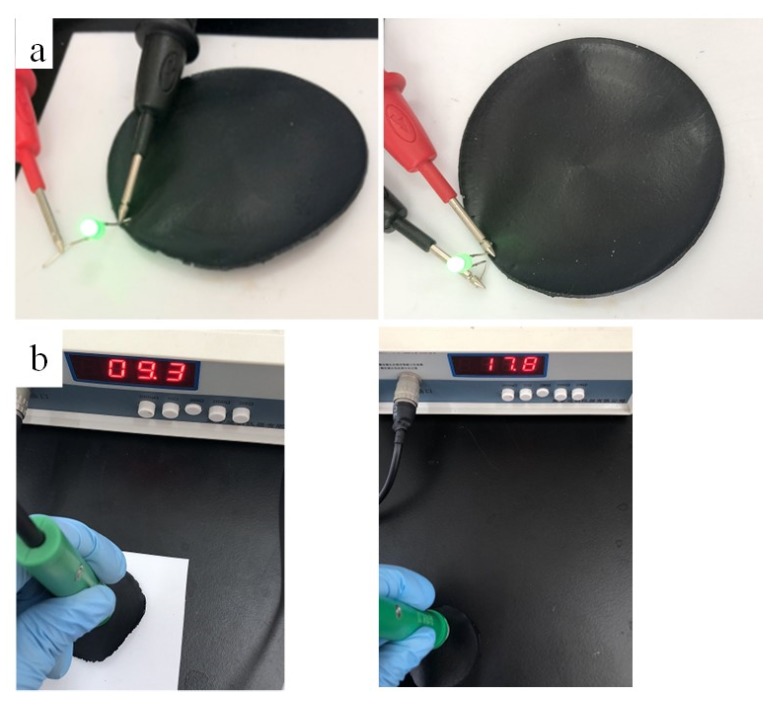
(**a**) Photos of light emitting diode devices on the conductive composite film without crack (left) and after healing (right), (**b**) testing photos of on the conductive composite film without crack (left) and after healing (right).

In order to examine the application of the self-healing conductive composite film, we fabricated light emitting diode(LED) devices on the conductive composite film without crack (Figure 16, left) and after healing (Figure 16, right), as shown in Figure 16. A 0.5 W LED lamp with series fixed on the surface of the conductive composite film without crack was lighted and after healed (Figure 16a, right). However, it should be noted that the sheet resistance of the self-healing composite film increases (tested on the same area), indicating there is not much graphene in the crack self-healing area. This is consistent with the previous discussion.

## 4. Conclusions

In summary, we fabricated graphene-thermopolyurethane (G-TPU) flexible conductive film by a blending method, and systematically investigated the electrical and thermal and self-healing properties of the G-TPU flexible conductive film by IR light and electricity. The experimental results demonstrate that the G-TPU composite films are conductive in the appropriate mass content of graphene in the composite film, but the conductivity of the composite film is related to the initial concentration of the TPU solution. Graphene improves the thermal stability of TPU. The thermal conductivity of composite film first increases and then decreases with the increase of the mass content of graphene in composite film. The composite film has good electro-thermal response performance and reaches a steady state temperature from room temperature in 70 s. The maximum steady state temperature of the composite films with the mass content of graphene of 3.0 wt%, 4.0 wt%, and 5.0 wt%, respectively, was 106.4 °C, 137.2 °C, and 169.3 °C, respectively. However, the temperature of the composite film heated by IR illumination first increases sharply, and then slowly, increases with the increase in the mass content of graphene in composite film. The temperature of the composite film with the mass content of graphene of 0.5 wt% reaches to 120.9 °C after heated for 60 s, that’s 1.7 times higher than that of the pure TPU film. The scratch on the composite film can be completely healed by electricity or IR light. The healing efficiency of the composite film healed using IR light is higher than that of using the electricity and the healing time is also shorter. Regardless of the self-healing method, the temperature of the self-healing is a very important factor. The self-healing conductive composite film still exhibited a good conductivity.

## Supplementary Materials

The following are available online at https://www.mdpi.com/2079-4991/10/4/753/s1, Figure S1: SEM image (a) and Raman spectrum (b) of graphene. Figure S2: Time-dependent temperature of the composite films with the mass contents of 0.5 wt% (a, b), and 4.0 wt% (c, d), respectively, and the initial concentration of TPU of 20% (a, c) and 30% (b, d) via IR light; Figure S3: Optical images of the scratch sample healed at 110 °C and 120 °C for different time using electricity.

## References

[1] Assel R., Thomas D.A.J., Sadeque R.K., Mohammadreza N.E., Matthew P.S., Russell A.H., Robert W.K., Marc P.Y.D., Jose M.-H. (2019). Selective Metallization of 3D Printable Thermoplastic Polyurethanes. IEEE Access.

[2] Pedrazzoli D., Manas-Zloczower I. (2016). Understanding phase separation and morphology in thermoplastic polyurethanes nanocomposites. Polymer.

[3] Xia H., Hashimoto Y., Ni Q.-Q. (2016). Electrically Triggered Actuation of Plasticized Thermoplastic Polyurethane Gels. Macromol. Mater. Eng..

[4] Alagi P., Choi Y.J., Hong S.C. (2016). Preparation of vegetable oil-based polyols with controlled hydroxyl functionalities for thermoplastic polyurethane. Eur. Polym. J..

[5] Huang L., Yi N., Wu Y., Zhang Y., Zhang Q., Huang Y., Ma Y., Chen Y. (2013). Multichannel and repeatable self-healing of mechanical enhanced graphene-thermoplastic polyurethane composites. Adv. Mater..

[6] Luan Y., Gao F., Li Y., Yang J., Hu Y., Guo Z., Wang Z., Zhou A. (2018). Healing mechanisms induced by synergy of Graphene-CNTs and microwave focusing effect for the thermoplastic polyurethane composites. Compos. Part A Appl. Sci. Manuf..

[7] Oh C.-R., Lee S.-H., Park J.-H., Lee D.-S. (2019). Thermally self-healing graphene-nanoplate/polyurethane nanocomposites via diels–alder reaction through a one-shot process. Nanomaterials.

[8] Cohades A., Branfoot C., Rae S., Bond I., Michaud V. (2018). Self-Healing Materials: Progress in Self-Healing Fiber-Reinforced Polymer Composites. Adv. Mater. Interfaces.

[9] Huynh T.-P., Sonar P., Haick H. (2017). Advanced Materials for Use in Soft Self-Healing Devices. Adv. Mater..

[10] Wu D.Y., Meure S., Solomon D. (2008). Self-healing polymeric materials: A review of recent developments. Progress in Polymer. Science.

[11] Min Y., Huang S., Wang Y., Zhang Z., Du B., Zhang X., Fan Z. (2015). Sonochemical transformation of epoxy-amine thermoset into soluble and reusable polymers. Macromolecules.

[12] Blaiszik B.J., Kramer S.L.B., Olugebefola S.C., Moore J.S., Sottos N.R., White S.R. (2010). Self-healing polymers and composites. Annu. Rev. Mater. Res..

[13] Wu S., Li J., Zhang G., Yao Y., Li G., Sun R., Wong C. (2017). Ultrafast self-healing nanocomposites via infrared laser and their application in flexible electronics. ACS Appl. Mater. Interfaces.

[14] Meng L.M., Yuan Y.C., Rong M.Z., Zhang M.Q. (2010). A dual mechanism single-component self-healing strategy for polymers. J. Mater. Chem..

[15] Bhargav A., Bell M.B., Cui Y., Fu Y. (2018). Polyphenylene Tetrasulfide as an Inherently Flexible Cathode Material for Rechargeable Lithium Batteries. ACS Appl. Energy Mater..

[16] Wang Z., Gao W., Zhang Q., Zheng K., Xu J., Xu W., Shang E., Jiang J., Zhang J., Liu Y. (2019). 3D-Printed Graphene/Polydimethylsiloxane Composites for Stretchable and Strain-Insensitive Temperature Sensors. ACS Appl. Mater. Interfaces.

[17] Altay B.N., Jourdan J., Turkani V.S., Dietsch H., Maddipatla D., Pekarovicova A., Fleming P.D., Atashbar M. (2018). Impact of Substrate and Process on the Electrical Performance of Screen-Printed Nickel Electrodes: Fundamental Mechanism of Ink Film Roughness. ACS Appl. Energy Mater..

[18] Huttunen O.-H., Happonen T., Hiitola-Keinänen J., Korhonen P., Ollila J., Hiltunen J. (2019). Roll-To-Roll Screen-Printed Silver Conductors on a Polydimethyl Siloxane Substrate for Stretchable Electronics. Ind. Eng. Chem. Res..

[19] Xu Y., Yang Y., Yan D., Duan H., Zhao G., Liu Y. (2019). Flexible and conductive polyurethane composites for electromagnetic shielding and printable circuit. Chem. Eng. J..

[20] Pang J.W.C., Bond I.P. (2005). A hollow fibre reinforced polymer composite encompassing self-healing and enhanced damage visibility. Compos. Sci. Technol..

[21] Jin H., Mangun C.L., Griffin A.S., Moore J.S., Sottos N.R., White S.R. (2014). Thermally stable autonomic healing in epoxy using a dual-microcapsule system. Adv. Mater..

[22] Lee Hia I., Chan E.-S., Chai S.-P., Pasbakhsh P. (2018). A novel repeated self-healing epoxy composite with alginate multicore microcapsules. J. Mater. Chem. A.

[23] Liu Y.-L., Chuo T.-W. (2013). Self-healing polymers based on thermally reversible Diels-Alder chemistry. Polym. Chem..

[24] Zeng C., Seino H., Ren J., Hatanaka K., Yoshie N. (2003). Bio-based furan polymers with self-healing ability. Macromolecules.

[25] Kuang X., Liu G., Dong X., Liu X., Xu J., Wang D. (2015). Facile fabrication of fast recyclable and multiple self-healing epoxy materials through diels-alder adduct cross-linker. J. Polym. Sci. Part A Polym. Chem..

[26] Davis D.A., Hamilton A., Yang J., Cremar L.D., Van Gough D., Potisek S.L., Ong M.T., Sottos N.R. (2009). Force-induced activation of covalent bonds in mechanoresponsive polymeric materials. Nature.

[27] Sottos N.R. (2014). Polymer mechanochemistry: Flex, release and repeat. Nat. Chem..

[28] Huang X., Qi X., Boey F., Zhang H. (2012). Graphene-based composites. Chem. Soc. Rev..

[29] Zhang Y., Li Y., Ming P., Zhang Q., Liu T., Jiang L., Cheng Q. (2016). Ultrastrong Bioinspired Graphene-Based Fibers via Synergistic Toughening. Adv. Mater..

[30] Fu Y.-X., He Z.-X., Mo D.-C., Lu S.-S. (2014). Thermal conductivity enhancement of epoxy adhesive using graphene sheets as additives. Int. J. Therm. Sci..

[31] Cai C., Zhang Y., Zou X., Zhang R., Wang X., Wu Q., Sun P. (2017). Rapid self-healing and recycling of multiple-responsive mechanically enhanced epoxy resin/graphene nanocomposites. RSC Adv..

[32] Li G., Xiao P., Hou S., Huang Y. (2019). Graphene based self-healing materials. Carbon.

[33] Debroya S., Miriyalaa V.P.K., Sekharb K.V., Acharyyab S.G., Acharyyaa A. (2016). Self healing nature of bilayer graphene. Superlattices Microstruct..

[34] Lin C., Sheng D., Liu X., Xu S., Ji F., Dong L., Zhou Y., Yang Y. (2019). Effect of different sizes of graphene on Diels-Alder self-healing polyurethane. Polymer.

[35] Guo Y., Zou D., Zhu W., Yang X., Zhao P., Chen C., Shuai M. (2019). Infrared induced repeatable self-healing and removability of mechanically enhanced graphene-epoxy flexible materials. RSC Adv..

[36] Kim J.T., Kim B.K., Kim E.Y., Kwon S.H., Jeong H.M. (2013). Synthesis and properties of near Ir induced self-healable polyurethane/graphene nanocomposites. Eur. Polym. J..

