# Supplementary material for: Electrical and Thermal and Self-Healing Properties of Graphene-Thermopolyurethane Flexible Conductive Films
Source: Nanomaterials (Basel). 2020 Apr 15;10(4):753. doi: 10.3390/nano10040753 (PMC7221931; doi:10.3390/nano10040753)
Supplement: Supplementary file 1 [file nanomaterials-10-00753-s001.pdf]

Supporting Information

Self-healing Graphene -Thermopolyurethane Flexible Conductive Films

Ke Wang [1,*], Zhimin Zhou[1,2], Jiahao Zhang[1], Jinyuan Tang[1], Peiyu Wu[1], Yuehui Wang[1,2,*] ,Yuzhen Zhao[3], and Yong Leng[4]

[1] Zhongshan Institute, University of Electronic Science and Technology of China, Zhongshan, Guangdong Province, P. R. China; Post Code: 528402; wkzsedu@126.com (K.W.); zjhzsedu@126.com（J. Z.）; tjyzsedu@126.com (J. T.); wpyzsedu@126.com (P.W.); wangzsedu@126.com (Y.W.)

[2] University of Material and Energy, University of Electronic Science and Technology of China, Chengdu 610054, China

[3] Department of Materials Science and Engineering, Tsinghua University, Beijing, P. R. China; Post Code: 100084; zhaoyz@mail.tsinghua.edu.cn (Y. Z.)

[4] Zhongshan Breathtex Speciality Material Co.,Ltd. Zhongshan, Guangdong Province, P. R. China; Post Code:528441. lybreathtex@126.com (Y.L)

* Correspondence: wangzsedu@126.com; Tel.: +86-760-8832-5402

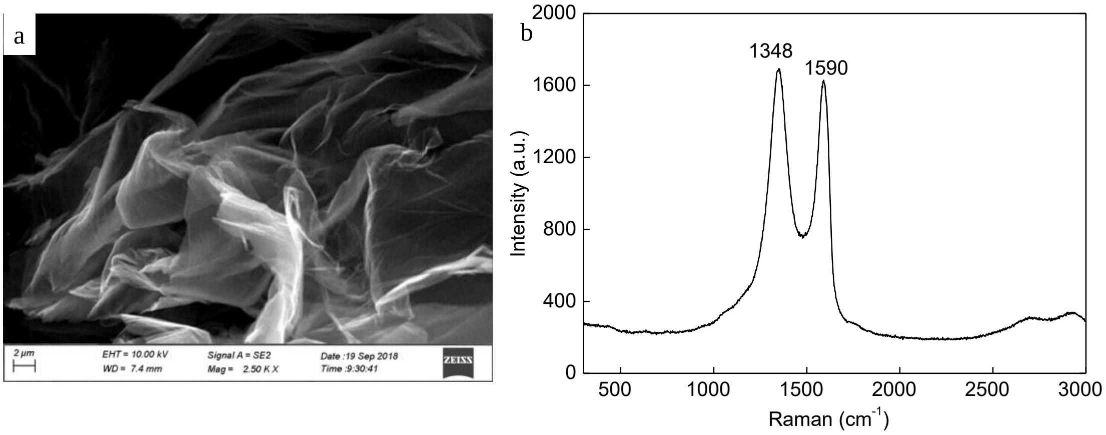

Figure S1 SEM image (a) and Raman spectrum (b) of graphene

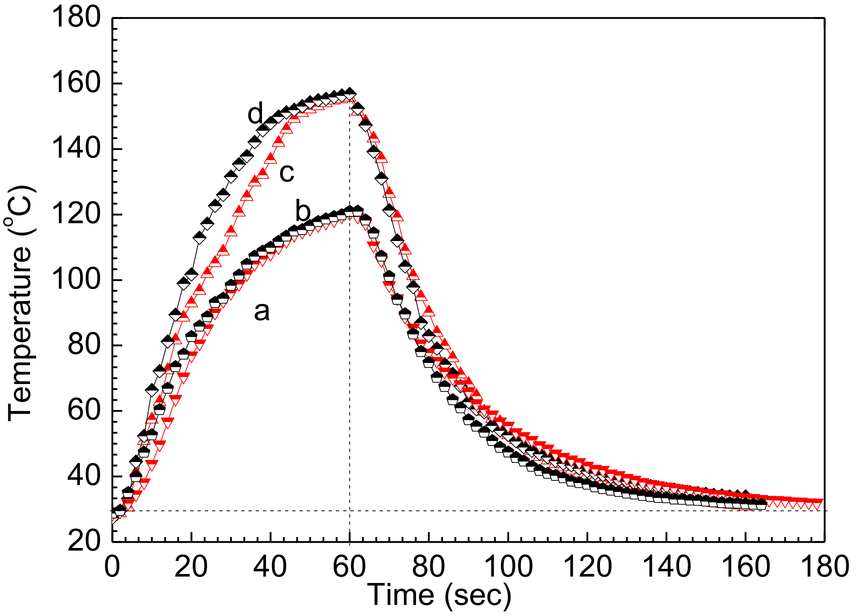

Figure S2 Time-dependent temperature of the composite films with the mass contents of 0.5 wt% (a, b), and 4.0 wt% (c, d), respectively, and the initial concentration of TPU of 20 % (a, c) and 30% (b, d) via IR light

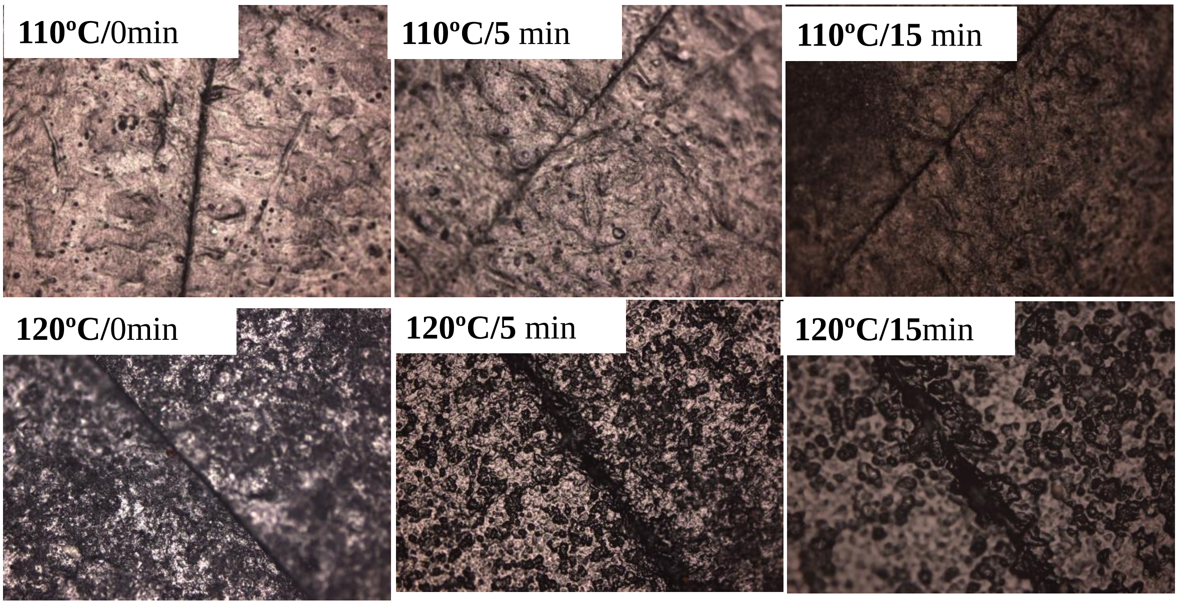

Figure. S3 Optical images of the scratch sample healed at 110 ºC and 120 ºC for different time using electricity.
